# Comprehensive Review of Geotechnical Engineering Properties of Recycled Polyethylene Terephthalate Fibers and Strips for Soil Stabilization

**DOI:** 10.3390/polym16131764

**Published:** 2024-06-21

**Authors:** Bisma Khalid, Fahad Alshawmar

**Affiliations:** 1 Department of Transportation Engineering and Management, University of Engineering and Technology, Lahore 54890, Pakistan; 2 Department of Civil Engineering, College of Engineering, Qassim University, Buraydah 51452, Saudi Arabia

**Keywords:** PET fibers, PET strips, polymer, geotechnical properties, soil stabilization, environmental impact, economic impact, sustainability

## Abstract

The waste management of plastic has become a pressing environmental issue, with polyethylene terephthalate (PET) being one of the major contributors. To address this challenge, the utilization of recycled PET fibers and strips in geotechnical engineering applications for soil stabilization has gained considerable attention. This review aims to provide a comprehensive study of the geotechnical engineering properties of recycled-PET-reinforced soils. The review examines various factors influencing the performance of PET-reinforced soils, including PET percent content, fiber length, and aspect ratio. It evaluates the mechanical properties, like shear strength, compressibility, bearing capacity, hydraulic behavior, and durability of recycled-PET-reinforced soils. The findings reveal PET reinforcement enhances shear strength, reduces settlement, and increases the bearing capacity and stability of the soil. However, it is observed that the incorporation of recycled PET fibers and strips does not lead to a significant impact on the dry density of the soil. Finally, an environmental and cost comparison analysis of recycled PET fibers and strips was conducted. This review serves as a valuable resource for researchers, engineers, and practitioners involved in the field, offering insights into the geotechnical properties of PET-reinforced soils and outlining future research directions to maximize their effectiveness and sustainability.

## 1. Introduction

Soil stabilization is a fundamental aspect of geotechnical engineering that involves improving the engineering properties of soils to enhance their load-bearing capacity, reduce settlement, and mitigate potential geotechnical hazards [1,2].

The strengthening mechanisms of soil are complex and multifaceted, involving various interactions between soil particles and reinforcements [3,4]. Particle rearrangement and interlocking, frictional resistance, cohesion and adhesion, and reinforcement through fibers or strips all contribute to soil strength [5]. These mechanisms are crucial for enhancing the mechanical properties of soil, such as shear strength, compressibility, and bearing capacity. The factors influencing soil strengthening mechanisms can be broadly categorized into soil-related properties and reinforcement-related properties. Soil-related properties include soil type and gradation, moisture content, density, and chemical and biological processes [6]. Reinforcement-related properties include fiber length, aspect ratio, material, and surface texture, as well as interface bonding between soil and reinforcement [7]. Understanding the interplay between these factors and mechanisms is essential for effective soil stabilization and reinforcement.

Traditionally, soil stabilization has relied on the use of conventional materials, such as cement, lime, and chemical additives, to modify soil characteristics [8,9]. However, with increasing environmental concerns and the need for sustainable construction practices, there has been a growing interest in utilizing recycled materials for engineering [10].

The use of recycled materials for soil stabilization offers several advantages, including environmental benefits and cost-effectiveness [11]. Recycling materials not only diverts waste from landfills but also reduces the consumption of virgin resources and lowers the overall carbon footprint associated with construction activities [12,13]. By incorporating recycled materials, geotechnical engineers can contribute to sustainable waste management practices while achieving the desired engineering performance.

A wide range of recycled materials can be used for soil stabilization in geotechnical engineering. These materials can include industrial by-products, such as fly ash from coal-fired power plants, slag from steel production, recycled aggregates, polyethylene terephthalate (PET), and crushed concrete [14,15]. Each material possesses unique properties that can be harnessed to improve soil characteristics and meet project requirements.

According to the researchers Mohee, et al. [16], plastic decomposition is not possible since plastic is not a biodegradable material. The traditional landfill methods for the disposal of plastic have negative effects on the environment [17,18]. Plastics, including PET, often contain additives and chemicals that can leach out when exposed to acidic environments, such as those found in landfills. This leaching process is accelerated as plastics degrade over time, releasing potentially harmful substances into the surrounding soil. As a result, soil organisms and ecosystems can be adversely affected by the presence of these chemicals, posing risks to environmental health [19]. Therefore, it is crucial to explore alternative methods for disposing of plastic materials. Plastic possesses several advantageous properties such as brittleness, strength, durability, resistance to chemical and corrosion, insect attacks, abrasion, and heat resistance as well as insulating properties [20,21]. To tackle the challenge of plastic waste management, one potential approach is to utilize plastic waste for soil stabilization.

Plastic waste constitutes a significant portion of the municipal waste stream, accounting for approximately 8–12% of total waste generated worldwide. Each year, countries across the globe produce an estimated 190 million tonnes of plastic waste [22]. Approximately 60% of the waste composition consists of construction and demolition (C&D) waste, while plastic waste makes up around 5% of the total waste. Within the plastic waste category, 3% comprises a combination of PET (polyethylene terephthalate), polyvinyl chloride (PVC), and high-density polyethylene (HDPE), with the remaining 2% consisting of other types of plastic materials [23].

PET fibers and strips are widely used plastic materials commonly found in beverage bottles, food containers, and packaging materials [24]. The disposal of PET waste poses a significant environmental challenge due to its non-biodegradable nature. However, recent studies have explored the possibility of utilizing recycled PET fibers and strips as a sustainable alternative for soil stabilization in geotechnical engineering applications [25]. The geotechnical engineering properties of recycled PET make it an attractive candidate for soil stabilization. PET has excellent tensile strength, durability, and resistance to chemicals, which are desirable characteristics for reinforcing and stabilizing soil [26,27]. Additionally, the abundance of PET waste presents an opportunity for recycling and transforming it into a valuable resource for geotechnical applications [28].

**Figure 1:**
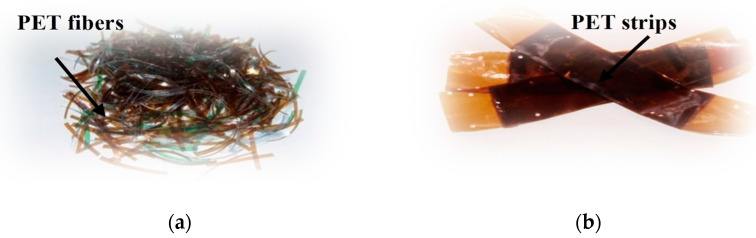
(**a**) PET fibers and (**b**) PET strips [29].

This comprehensive review aims to provide an in-depth analysis of the geotechnical engineering properties of recycled PET fibers and strips for soil stabilization as shown in Figure 1. The review will cover various aspects, including the effect of recycled PET on soil mechanical properties. Furthermore, the potential benefits and drawbacks of utilizing recycled PET fibers and strips for soil stabilization will be critically assessed, considering factors such as environmental impact and compatibility with existing geotechnical practices. Overall, this review aims to contribute to the advancement of sustainable soil stabilization techniques by highlighting the geotechnical engineering properties of recycled PET. The findings of this review will be valuable for researchers, engineers, and practitioners involved in geotechnical engineering, providing them with a comprehensive reference for the utilization of recycled PET fibers and strips as a viable solution for soil stabilization, while promoting the principles of environmental sustainability.

## 2. Materials and Methods

An extensive literature search was performed utilizing academic databases including Web of Science, Scopus, and Google Scholar from September 2023 to January 2024. Relevant publications, conference papers, and reports published up to the current date were identified by using keywords such as “recycled PET fibers in geotechnical engineering”, “PET-reinforced soils”, and similar variations. From this search, we identified more than 140 relevant papers published in the last decade. We filtered these papers based on relevance and quality, focusing on peer-reviewed articles and high-impact journals. The selection criteria included studies that provided empirical data, comprehensive reviews, or significant advancements in the understanding of soil strength mechanisms and factors. After filtering, the geotechnical parameters of soils reinforced with PET were obtained from the chosen literature. The analyzed data were compiled to detect trends and patterns in the performance of soils reinforced with PET. This encompassed data regarding the PET content; fiber length; aspect ratio; mechanical properties such as shear strength (ASTM D4767-11) [30], Atterberg limits (ASTM D4318) [31], compressibility (ASTM D2166) [32], and bearing capacity (ASTM D1883) [33]; hydraulic behavior (ASTM D5084) [34]; and other relevant variables that affect soil behavior. The research findings were combined and presented in an organized way, highlighting the efficacy of PET fibers and strips in soil stabilization and their compatibility with various soil types. This analysis aimed to provide engineers and academics with crucial insights to promote sustainable practices in the field of geotechnical engineering.

## 3. Properties of PET

### 3.1. Structural Properties of PET

**Figure 2:**
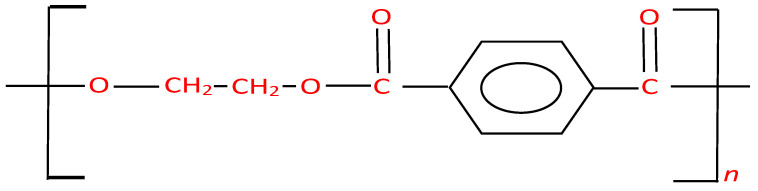
Chemical structure of PET.

PET, a thermoplastic polymer, is widely recognized for its recyclability and has gained global significance due to its extensive range of plastic applications. The chemical structure of PET, represented in Figure 2, depicts the arrangement of its repeating units, with ‘n’ representing the number of these units. PET fibers and strips possess several important structural properties. The ethylene glycol units (-O-CH_2_-CH_2_-O-) are connected by ester linkages to the terephthalic acid units (-C_6_H_4_-CO-O-), forming a linear chain [35]. The ester linkages are formed through a condensation reaction between the hydroxyl groups of ethylene glycol and the carboxyl groups of terephthalic acid [36]. This chemical structure gives PET its notable properties. The presence of the ester linkages contributes to PET’s high strength, durability, and thermal stability. The polymer chains are closely packed, creating a rigid and crystalline structure, which further enhances PET’s mechanical properties [37]. The chemical structure of PET also enables its excellent barrier properties, making it resistant to moisture, gases, and chemicals. This property makes PET a preferred material for packaging applications, as it helps to protect and preserve the contents of various products [38]. Furthermore, the presence of the aromatic ring in the terephthalic acid units enhances PET’s chemical resistance and makes it less susceptible to degradation from ultraviolet (UV) light exposure, providing it with good UV stability [39].

### 3.2. Thermal, Mechanical, and Physiochemical Properties of PET

In geotechnical engineering, semi-crystalline PET and amorphous PET offer distinct advantages and applications. Semi-crystalline PET, characterized by its ordered molecular structure, provides superior mechanical strength and stiffness, making it suitable for reinforcing soil in applications requiring high load-bearing capacities, such as embankments and retaining walls. Its strength enhances soil stability and durability. Conversely, amorphous PET lacks a defined molecular arrangement, offering flexibility and transparency. In geotechnical engineering, amorphous PET is often used in erosion control mats or geotextiles, where flexibility and ease of installation are paramount. While semi-crystalline PET ensures structural integrity, amorphous PET provides adaptability and ease of use, catering to diverse geotechnical engineering needs [40].

Polyethylene terephthalate (PET) is a thermoplastic polymer with distinct thermal, mechanical, and physiochemical properties, as shown in Table 1. A key property to consider is the glass transition temperature (T_g_) of the polymer, which signifies the temperature at which the amorphous sections of the material shift from a rigid, glass-like state to a more pliable, rubbery state. For PET, the T_g_ is typically around 60 to 80 °C [41]. The maximum service temperature (T_max_) of PET denotes the uppermost temperature at which the material can endure continuous exposure without experiencing substantial degradation. This temperature can vary depending on the specific formulation and processing conditions. In terms of molding, PET requires a specific temperature range for processing known as the mold temperature (T_mould_). The T_mould_ typically falls within the range of 120 to 150 °C for semi-crystalline PET [42], allowing the polymer to melt and flow easily into the desired shape within the mold. Finally, PET has a melting temperature (Tm), which is the temperature at which the crystalline regions of the polymer melt and the material undergoes transitions from a solid state to a liquid state. The T_m_ of PET is relatively high, ranging from approximately 240 to 260 °C for semi-crystalline PET [42].

PET exhibits several important mechanical properties. One of the key properties of PET is its Young’s modulus, denoted by E, which represents its stiffness or resistance to deformation under an applied force. PET typically has a Young’s modulus ranging from 2 to 4 GPa, depending on the specific grade and processing conditions [43]. Another important mechanical property of PET is its elongation at break, represented by ε_b_. Elongation at break is a measure of the material’s ability to withstand deformation before fracturing or breaking. PET generally exhibits an elongation at a break of around 50–100%, meaning it can stretch to about 1.5 to 2 times its original length before failure [44]. The maximum stress (σ_max_) is another significant mechanical property of PET. It represents the maximum force or load that PET can withstand before fracturing or yielding. The maximum stress of PET typically ranges from 50 to 100 MPa, depending on the grade and processing conditions [45].

PET has several important physicochemical properties. Density is a key property of PET and is typically around 1.38 g/cm^3^ [46]. This relatively high density contributes to its strength and durability, making it suitable for various applications. PET exhibits low permeability to gases such as carbon dioxide (CO_2_) and oxygen (O_2_) [47]. At 25 °C, the permeability of PET to CO_2_ is relatively low, indicating its ability to act as a barrier to prevent gas transmission. PET also has low oxygen permeability, which makes it useful for packaging applications where oxygen barrier properties are essential to protect the contents [48]. Transparency is another notable property of PET. PET is a highly transparent material, allowing for excellent clarity and visibility. PET’s transparency is particularly advantageous for packaging applications where product visibility is important, such as in beverage bottles or food containers [49,50].

## 4. Polymerization and Conventional Recycling Process of PET

### 4.1. Polymerization Process of PET

**Figure 3:**
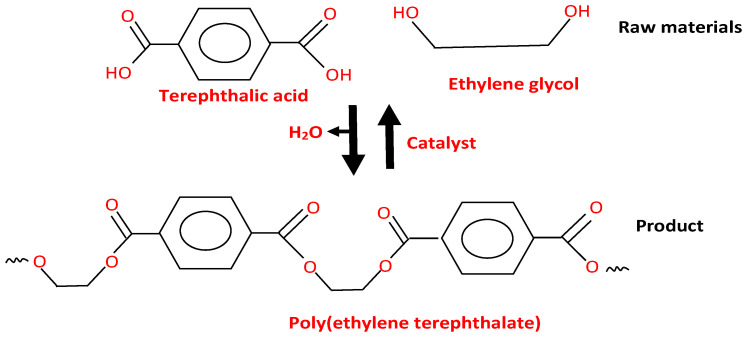
Polymerization process of PET.

The polymerization process of PET begins with the preparation of its monomers, purified terephthalic acid (PTA), and ethylene glycol (EG), as shown in Figure 3 [51]. These raw materials undergo an esterification step, where they are combined in the presence of a catalyst, usually antimony trioxide, and subjected to a high-temperature reaction. This produces a monomer called bis(2-hydroxyethyl) terephthalate (BHET) [52]. The BHET monomer then goes through pre-polymerization, where it is heated at a lower temperature in the presence of a metal-based catalyst. This step results in a low-molecular-weight polymer. The prepolymer is further polymerized through a solid-state polycondensation (SSP) process. It is heated under vacuum conditions to remove impurities and fed into SSP reactors [53]. In these reactors, the prepolymer undergoes solid-state polymerization, which gradually increases its molecular weight and viscosity. The process continues until the desired properties are achieved. Once polymerization is complete, the molten PET is extruded into long strands, cooled, and then cut into small pellets or granules, which can be further processed into various products using techniques like injection molding or extrusion [54]. While specific variations may exist among manufacturers, these general steps provide an overview of the PET polymerization process.

### 4.2. Conventional PET Recycling Process

**Figure 4:**
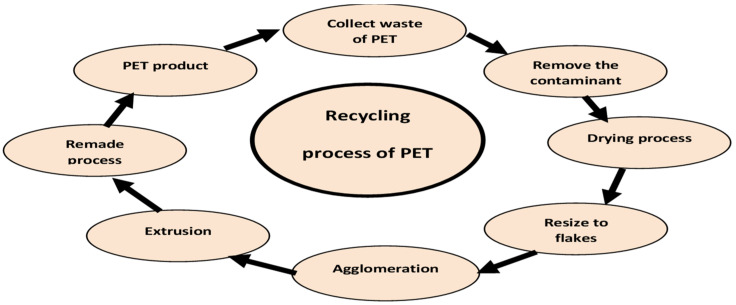
Conventional recycling process of PET [62].

The conventional recycling process of PET is shown in Figure 4. The conventional PET recycling process begins with the collection of used PET products, such as plastic bottles, from recycling bins or dedicated collection points [55,56]. Once collected, the PET items undergo sorting to separate them from other types of plastics. This ensures the purity of the recycled PET material. Following sorting, the PET is thoroughly cleaned to eliminate any remaining impurities, including labels, caps, and residual liquids [57]. Cleaning methods can involve washing with water and detergent or using specialized equipment with friction, heat, or chemicals [58]. After cleaning, the PET is crushed or shredded into small flakes, which increases its surface area for easier handling and further processing. The flakes then undergo a decontamination process to remove any remaining impurities or residues, ensuring the quality and safety of the recycled PET material [59]. The next step involves melting the PET flakes to form molten PET through extrusion. This process involves heating the flakes until they become a viscous liquid. The molten PET can then be molded into pellets, fibers, or sheets, depending on the intended use. The molten PET may undergo additional processing, such as solid-state polymerization, to enhance its quality and performance. Solid-state polymerization involves subjecting the molten PET to heat and pressure, which improves its molecular structure, making it stronger and more suitable for specific applications. Finally, recycled PET material, in the form of pellets, fibers, or sheets, is readily available as a raw material for the manufacturing of diverse products including polyester fabrics, carpets, packaging materials, and new PET bottles. Through this conventional recycling process, PET waste is effectively reduced, valuable resources are conserved, and a more sustainable approach to plastic consumption and production is promoted [60,61].

## 5. Distribution and Placement of Recycled PET in Soil

The distribution and placement of PET fibers and strips in the soil play a crucial role in soil stabilization. To effectively utilize PET for soil stabilization, several techniques are employed. Firstly, the soil is prepared by ensuring proper compaction and uniformity. Then, PET fibers and strips are evenly distributed throughout the soil using specialized equipment, such as spreaders or mixers [63]. Depending on the application, the PET fibers and strips may be incorporated at different depths within the soil profile. Techniques such as soil mixing or injection may be employed for deeper placement. Once distributed, the soil–PET fiber and strip mixture is compacted to ensure proper bonding and integration of the fibers and strips. Additionally, geotextiles or geogrids made from PET fibers and strips can be directly placed onto the soil surface or buried within the soil to provide reinforcement [64]. Overall, meticulous attention to the application and placement of PET is essential for optimizing soil stabilization and improving soil properties effectively.

## 6. Applications of Recycled PET Fibers in Soil Stabilization

Recycled PET fibers have numerous applications in geotechnical engineering, contributing to both environmental sustainability and improved performance [65,66]. One significant application of PET fibers classified as geogrids is soil stabilization, where these fibers are incorporated into the soil to enhance its strength and resistance against erosion, settlement, and shear forces. They can also be used in retaining walls and erosion control systems, providing structural support and preventing soil erosion [67]. Furthermore, they find utility in land reinforcement, strengthening weak areas, and improving load-bearing capacity in infrastructure projects [68]. Utilizing recycled PET fibers in geotechnical engineering helps to reduce plastic waste and embrace a more sustainable approach to construction.

### 6.1. Influence of Recycled PET Fibers on Soil’s Stress–Strain Characteristics

**Figure 5:**
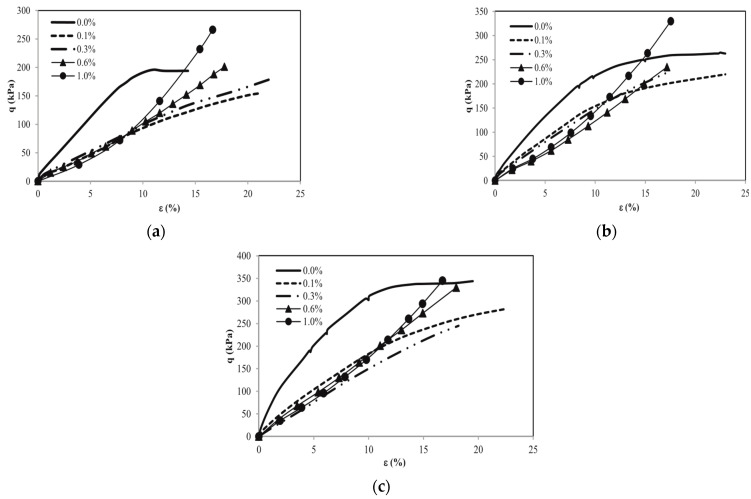
Stress–strain curves for: (**a**) σ_c_ = 61.85 kPa, (**b**) σ_c_ = 123.70 kPa, and (**c**) σ_c_ = 185.56 kPa. Reprinted from [70], Copyright (2023) with permission from Elsevier.

The stress–strain characteristics of PET-fiber-stabilized soil exhibit distinct behavior compared to unstabilized soil [69]. The behavior of reinforced and unreinforced specimens was examined by Botero, et al. [70] by plotting stress–strain curves with the help of triaxial test results. Figure 5 demonstrates that the stress–strain responses vary depending on the quantity of recycled PET fiber and the level of confining pressure (σ_c_) in silty soil. The stress–strain behavior of the reinforced soil specimen, utilizing recycled PET fibers ranging from 0.1 to 1%, demonstrates a linear response. Prior to the strain range of 10–15%, the soil reinforced with PET fibers exhibits non-plastic behavior. Beyond this range, the soil shows plastic behavior but does not display an increase in soil resistance until failure occurs. Therefore, the addition of recycled PET fibers has a noticeable effect on the stress–strain behavior of the soil. Specifically, specimens with fiber percentages of 0.60% and 1.00% exhibit greater resistance increments near 10% strain. According to researchers Mariri, et al. [71], recycled PET fibers into a mixture of zeolite, cement, and loess resulted in an increase in the strain at failure. The addition of recycled PET fibers in soil has been observed to influence the linear stress–strain response. This enhancement in the stress–strain response is attributed to the reinforcing effect of the fibers, but variation in the stress–strain behavior may occur depending on the soil type used [72,73,74].

### 6.2. Influence of Recycled PET Fibers on Soil’s Liquid, Plastic, and Shrinkage Limits

**Figure 6:**
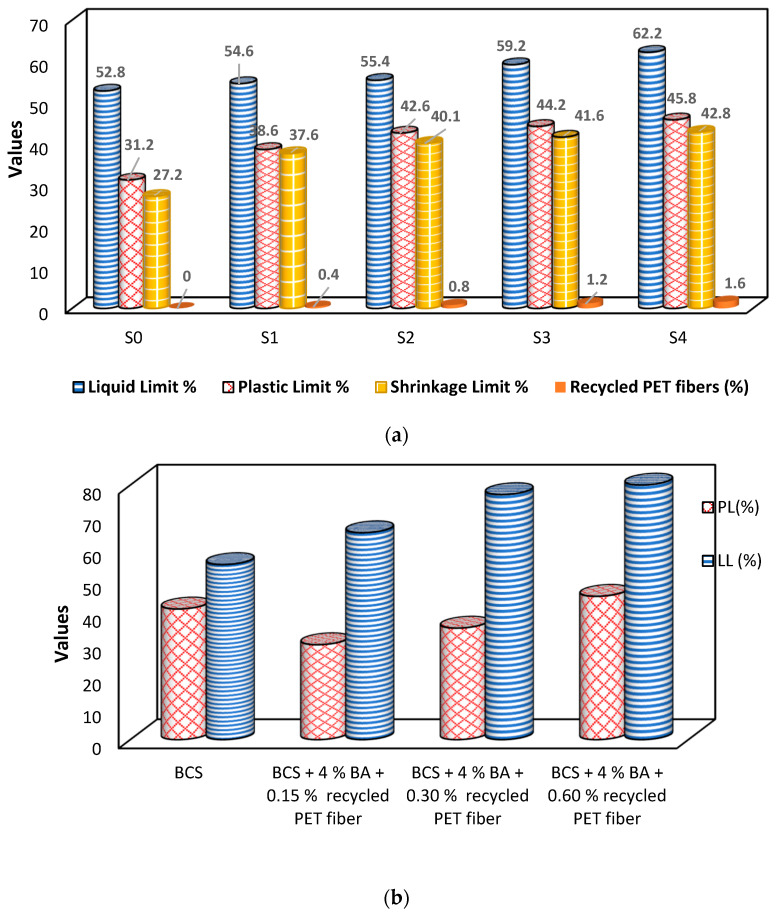
Variation caused by recycled PET fibers in liquid, plastic, and shrinkage limits of soil: (**a**) [14] and (**b**) [77]. Abbreviations: S0: soil sample with 0% PET fiber; S1: soil sample with 0.4% PET fiber; S2: soil sample with 0.8% PET fiber; S3: soil sample with 1.2% PET fiber; S4: soil sample with 1.6% PET fiber; BA: bagasse ash; BCS: black cotton soil.

The liquid, plastic, and shrinkage limits of the modified soil increase as the percentage content of recycled PET fibers increases, as shown in Figure 6. For soil samples S0, S1, S2, S3, and S4, the addition of recycled PET fibers with fly ash leads to liquid limit (LL) increases by factors of 1.03, 1.05, 1.12, and 1.18, respectively. These alterations facilitate the substitution of soil grains with recycled PET fibers, resulting in enhanced continuity in the soil. The hydrophobic nature of recycled PET fibers prevents moisture absorption, contributing to the higher liquid limit observed in the reinforced soil [14]. According to the researchers Kinjal, et al. [75], the liquid limit of soil decreases as the content of recycled polyester fibers increases in clayey soil. However, the variation in liquid limit becomes significantly less after a fiber content of 0.5% is reached. Changizi and Haddad [76] found that the addition of 0.5% recycled PET fibers leads to a 1.04-fold increase in the value of the liquid limit. According to the research conducted by Arya, et al. [77] on black cotton soil, the addition of bagasse ash and recycled PET fiber results in an increase in the liquid limit value of the soil, as shown in Figure 6.

The plastic limit (PL) of the reinforced soil samples (S0, S1, S2, S3, and S4) shows an increase by factors of 1.24, 1.37, 1.42, and 1.47, respectively. The plastic index (PI) value of the soil decreases as the PET fiber content is increased in conjunction with fly ash. The PI of soil reinforced by PET fibers decreases by factors of 0.74, 0.59, 0.69, and 1.03, respectively, for different percentages of recycled fiber in conjunction with fly ash [14]. Kinjal, Desai and Solanki [75] found that increasing the percentage of recycled PET fibers in the clayey soil results in an increase in the plastic limit, but after a fiber content of 0.5% is reached, the plastic limit starts to decrease. Similar outcomes were observed by Fauzi, et al. [78]. For recycled fiber percentages of 0.5%, 1.0%, and 1.5%, the inclusion of fibers results in increases in the plastic limit by factors of 1.29, 1.42, and 1.44, respectively [76]. The study performed by Arya, Patel, Bharti, Shukla and Hurukadli [77] found similar performance by adding recycled PET fibers to black cotton soil, as shown in Figure 6. The shrinkage limit of the soil samples (S0, S1, S2, S3, and S4) is increased by factors of 1.38, 1.47, 1.53, and 1.57, respectively, with the presence of recycled PET fibers in conjunction with fly ash [14]. The same result was found by Kinjal, Desai and Solanki [75] up to a 0.5% increase in recycled fiber contents, as shown in Figure 6. Therefore, the addition of recycled PET fibers in clayey soil in conjunction with fly ash proves to be an effective way of enhancing tensile strength and their ability to withstand volumetric changes in soil [14]. The study performed by Harianto, et al. [79] revealed that increasing the fiber content to 1.0% resulted in a significant 20% increase in the shrinkage limit compared to no fiber addition. However, at a higher fiber percent of 1.2%, the shrinkage limit slightly decreased. This can be attributed to the filling of soil voids by fibers at 1.2%, which led to reduced contact between soil particles and recycled PET fibers, resulting in less resistance and a lower shrinkage limit. Thus, an increase in the percentage of fiber causes an increase in the shrinkage limit of soil [76].

### 6.3. Influence of Recycled PET Fibers on Soil’s Dry Density

Modified Proctor test results indicate that as the percentage of recycled PET fibers increases, there is a minimal variation in both the moisture content and maximum dry density of the soil [14,80,81]. These results were similar to the research conducted by Yadav and Tiwari [82]. According to the findings of Miller and Rifai [80], the variations in the maximum dry density and the optimum value of moister content, resulting from the addition of fibers, are less than 5%. These variations are considered insignificant in terms of compaction. In other words, the addition of recycled fibers does not have a significant influence on the maximum dry density and optimum moisture content values, suggesting that compaction efforts may not be significantly affected by the fiber addition [83].

### 6.4. Influence of Recycled PET Fibers on Soil’s Normal–Shear Stress Characteristics

**Figure 7:**
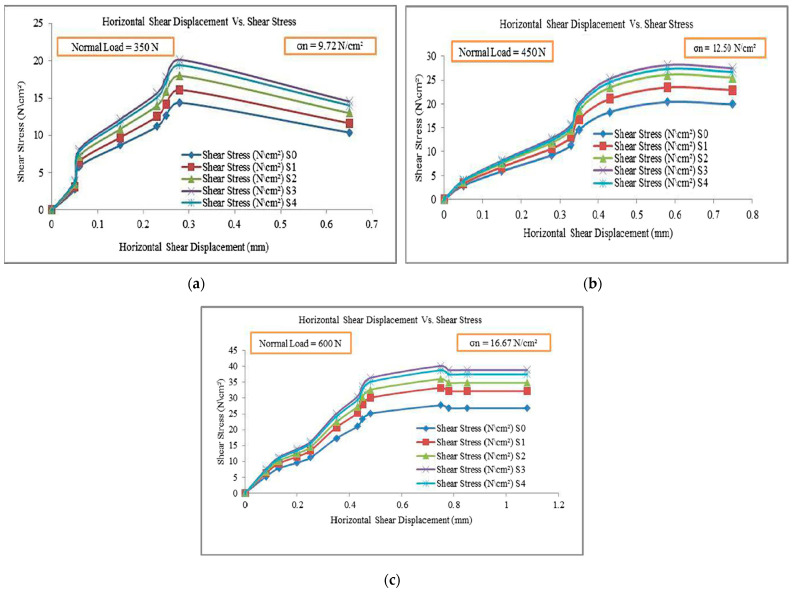
Shear stress vs. horizontal shear displacement at: (**a**) σ_n_ = 9.72 N/cm^2^, (**b**) σ_n_ = 12.50 N/cm^2^, and (**c**) σ_n_ = 16.67 N/cm^2^. Reprinted from [14], Copyright (2023) with permission from Elsevier.

According to the research conducted by Mishra and Gupta [14], with an increase in the percentage of recycled PET fibers with fly ash, there is an observed enhancement in the strength behavior of the soil. The maximum rise in peak strength is observed at different normal stress (σ_n_) levels from 9.72 N/cm^2^ to 16.67 N/cm^2^, as shown in Figure 7. For the soil samples S0, S1, S2, S2, S3, and S4, the peak shear stress increases by factors of 1.12, 1.25, 1.40, and 1.35, respectively, at a normal stress of 9.72 N/cm^2^. Similar trends are observed at higher normal stress levels. However, when the PET fiber percentage exceeds 1.2%, the peak strength of the reinforced soil starts to decrease. This decrease is due to the excessive fiber-to-soil ratio, leading to inefficient interlocking and reduced load transfer mechanisms. At a fiber percentage beyond 1.2%, the contact between PET fibers and the mixture decreases, and the fiber-to-fiber contact becomes dominant. This relative volume engaged by the recycled PET fibers is a possible cause for the strength reduction. The serration in Figure 7 shear test data is the result of “stick–slip” behavior during the shearing process. This phenomenon happens when soil particles connect and then break away, causing shear stress to fluctuate. Soil heterogeneity, strain localization, instrument sensitivity, frictional sliding, and particle rearrangement all play a role in this behavior [84,85]. Understanding these parameters allows for a more accurate interpretation of shear test data, which provides insight into soil mechanical properties under shear stress.

**Figure 8:**
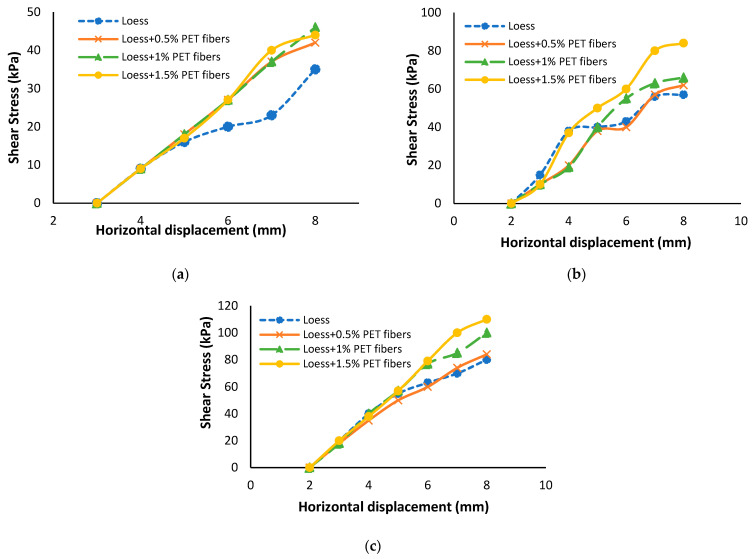
Shear stress vs. horizontal shear displacement at: (**a**) σ_n_ = 50 kPa, (**b**) σ_n_ = 100 kPa, and (**c**) σ_n_ = 150 kPa [81].

**Figure 9:**
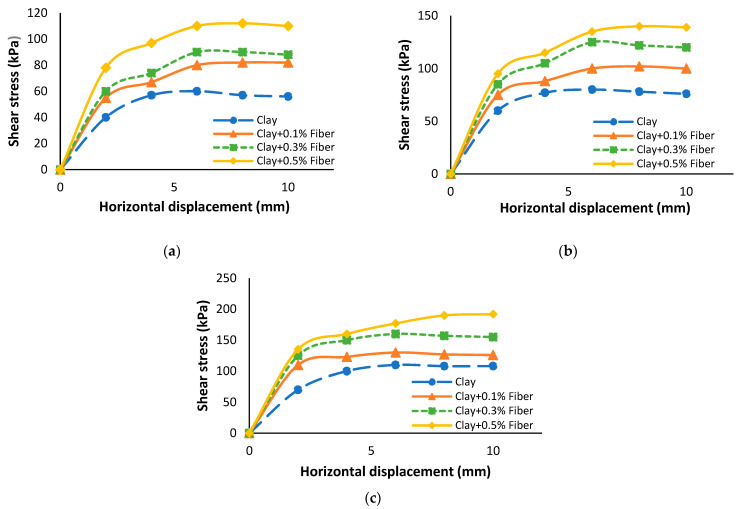
Shear stress vs. horizontal shear displacement at: (**a**) σ_n_ = 100 kPa, (**b**) σ_n_ = 200 kPa, and (**c**) σ_n_ = 300 kP [76].

Interlocking between the particles of soil and the PET fiber surface plays a significant role in improving shear strength. Changizi and Haddad [72] observed that the inclusion of 0.5% recycled PET fiber resulted in a significant increase in the shear stress value from 111 kPa to 200 kPa when compared to natural clay. This increase signifies a remarkable enhancement in shear strength, amounting to an 80% improvement due to the addition of recycled PET fibers. Sarli, Hadadi and Bagheri [81] study found that the addition of 1.5% recycled polyester fiber increased the peak shear stress from 55 kPa to 121 kPa, resulting in a 53% increase in shear strength of soil as compared to natural loess. Moreover, when the fiber percent content is 0.5% and 1%, peak shear stresses increased by 14% and 29% in loess soil, respectively. However, at a higher fiber percentage of 1.5%, the rise in shear strength value was only 53%, as shown in Figure 8. Based on the stress–displacement behavior observed in Figure 9, it can be inferred that the peak strength of recycled fiber-reinforced clayey soil was generally reached at larger horizontal displacements compared to the unreinforced soil in most of the specimens examined [76]. The results of Kholghifard and Amini Behbahani [86] obtained from clayey sand were similar to the research of [76,87].

### 6.5. Influence of Recycled PET Fibers on Soil’s California Bearing Ratio

**Figure 10:**
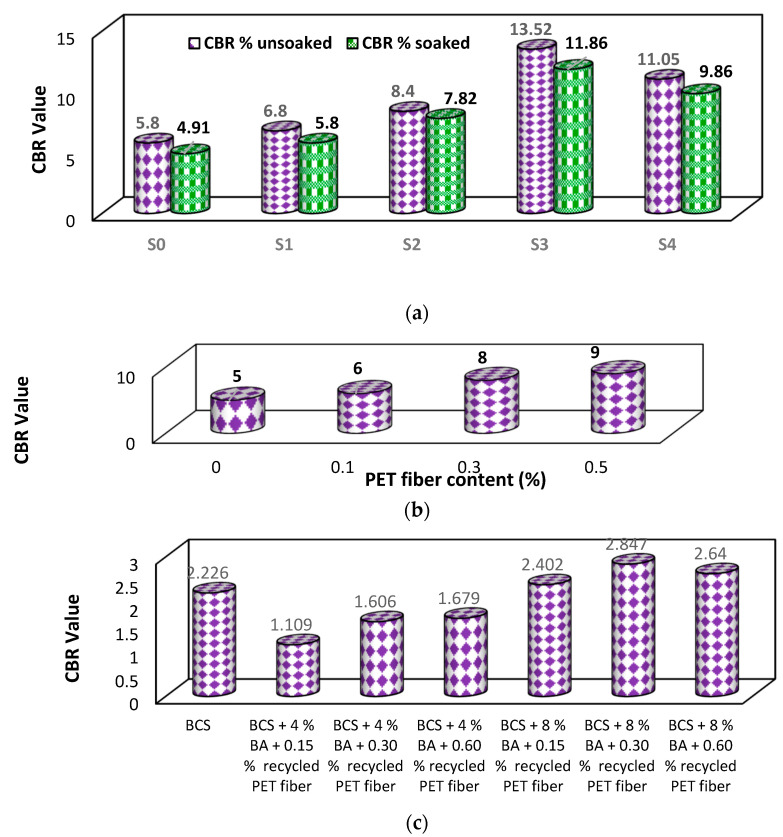
Variation in CBR value with the use of recycled PET fibers: (**a**) [14], (**b**) [76], and (**c**) [77].

According to Mishra and Gupta [14], an increase in the percentage of recycled PET fibers leads to an increase in the CBR (California Bearing Ratio) value, as depicted in Figure 10a. The maximum CBR value is achieved at a fiber percentage of 1.2%. However, after this peak is reached, further increases in the PET fiber percentage result in a decrease in the value of the CBR, both for soaked and unsoaked conditions. The addition of recycled PET fibers in soil improves the CBR by enhancing the interfacial friction and reducing deformation, resulting in increased strength and performance. The results obtained by Changizi and Haddad [76] demonstrate that the inclusion of fibers leads to an increase in the CBR. The study further reveals that the CBR values show an upward trend with increasing fiber content up to 0.3%. However, as the percentage of fiber continues to increase beyond this point, the incremental improvement in CBR values diminishes, as shown in Figure 10b. Therefore, an increase in recycled PET fiber content has a significant impact on the CBR value of soil [88,89]. Arya, Patel, Bharti, Shukla and Hurukadli [77] revealed that the inclusion of 4% bagasse ash along with recycled PET fiber led to a reduction in the value of the CBR of black cotton soil (BCS). However, with a further increase in bagasse ash to 8%, the CBR value reached its maximum at 2.847 with a fiber content of 0.3%, as depicted in Figure 10c.

### 6.6. Influence of Recycled PET Fibers on Soil’s Indirect Tensile Strength

**Figure 11:**
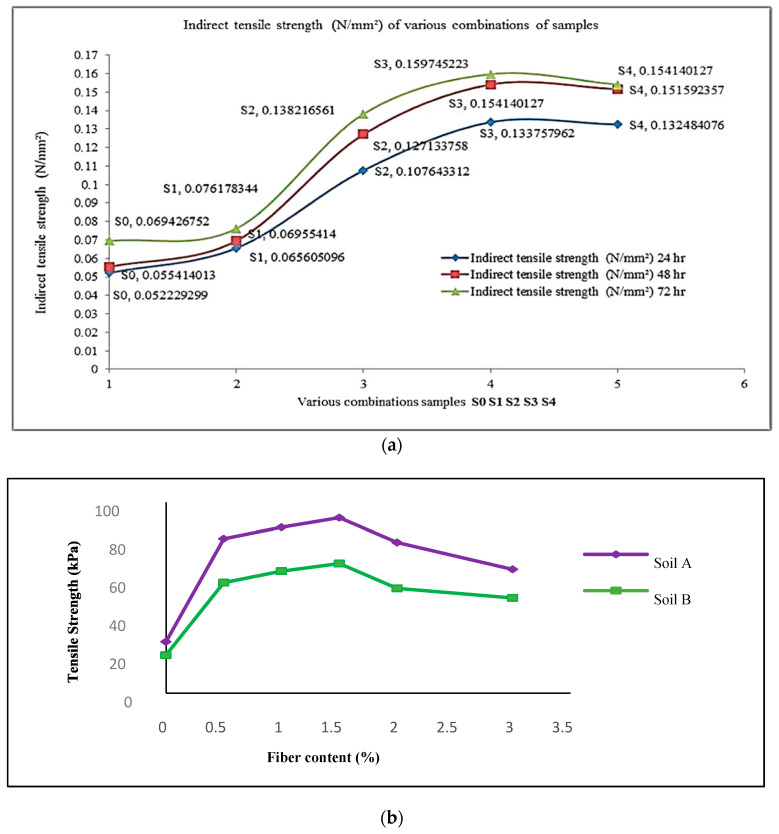
Variation caused by recycled PET fibers in CBR value of soil. (**a**) Reprinted from [14], Copyright (2023) with permission from Elsevier; (**b**) [90].

The addition of 1.2% recycled PET fibers in different soil types yields better performance. The addition of recycled PET fibers minimizes the formation of tension cracks through a bridge effect. The interfacial friction between recycled PET fibers and mix particles increases, leading to increased strength. However, when the PET fiber content exceeds 1.2%, the contact between the PET fibers and particles decreases, and fiber-to-fiber interaction becomes dominant, as shown in Figure 11a. This results in a reduction in strength, possibly due to the increased volume occupied by the fibers [14,87]. According to Tafti and Emadi [90], when the PET fiber content is increased up to 1.5%, there is an observed increase in the indirect tensile strength of poorly graded soils. However, beyond this fiber content, the strength begins to decrease with further increases in the PET fiber content, as shown in Figure 11b.

### 6.7. Influence of Recycled PET Fibers on Soil’s Crack Reduction Ratio

Cracked area refers to the surface area of cracks on the soil sample after it has undergone stress or drying [91]. To calculate this, images of the cracked soil surface are taken, and image analysis software is used to measure the total area occupied by the cracks. This involves processing the images to distinguish between cracked and uncracked areas and then summing the areas of all detected cracks [92]. This measurement helps in assessing how different contents of recycled PET fibers influence the reduction in soil cracking. A smaller cracked area indicates that the fibers are effective in enhancing soil stability and integrity by reducing the formation and propagation of cracks [93].

**Figure 12:**
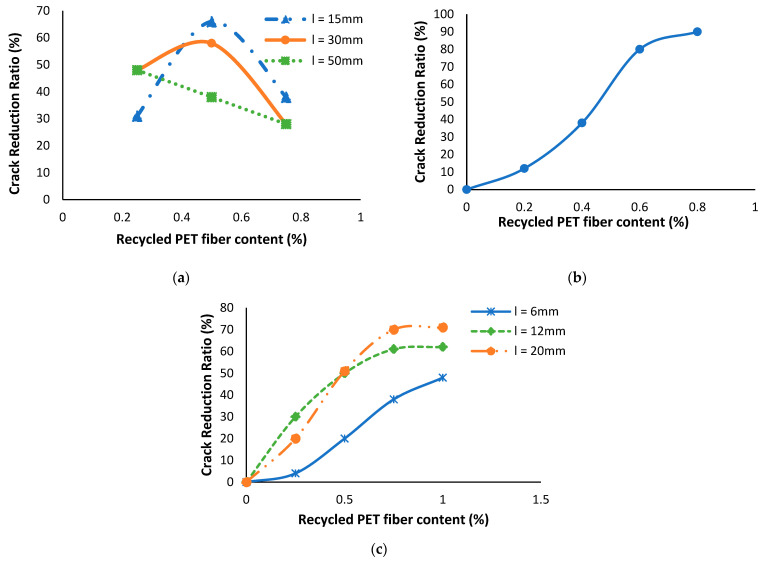
Variation in crack reduction ratio with different recycled PET fiber contents: (**a**) [94], (**b**) [80], and (**c**) [95].

Chaduvula, et al. [94] examined the effect of recycled PET fibers in expansive soil. According to Chaduvula, Viswanadham and Kodikara [94], fiber reinforcement results in a reduction in the cracked area ranging from 26% to 66% in comparison to the soil specimen without reinforcement. The specimen with a PET fiber content of 0.5% and fiber length of 15 mm demonstrates the highest reduction in cracks, as shown in Figure 12a. This reduction can be attributed to the enhancement of the clay mass’s tensile strength due to the addition of fibers. The findings of Miller and Rifai [80] indicate that increasing the recycled fiber content from 0.2% to 0.8% resulted in a significant improvement in crack reduction. Specifically, the crack reduction increased from 12.3% to 88.6% across the range of fiber content, as shown in Figure 12b. Olgun [95] research indicates that the crack reduction ratio increases with an increase in fiber content, as shown in Figure 12c. However, the study also found that the crack reduction shows only a minimal increase after a PET fiber content of 0.75% is reached. Harianto, Hayashi, Du and Suetsugu [79] conducted tests on the crack intensity factor (CIF) of soil samples and presented that there was a decrease in the volumetric change of compacted soil samples with the addition of recycled PET fiber. In other words, the inclusion of fiber reduced the extent of shrinkage and cracking in the soil, as indicated by the decreased volume change [96,97]. Gupta, et al. [98] reported a significant reduction in cracking, reaching up to 89 percent compared to the control specimen, through the incorporation of recycled polyester fiber.

### 6.8. Influence of Recycled PET Fibers on Soil’s Hydraulic Conductivity

**Figure 13:**
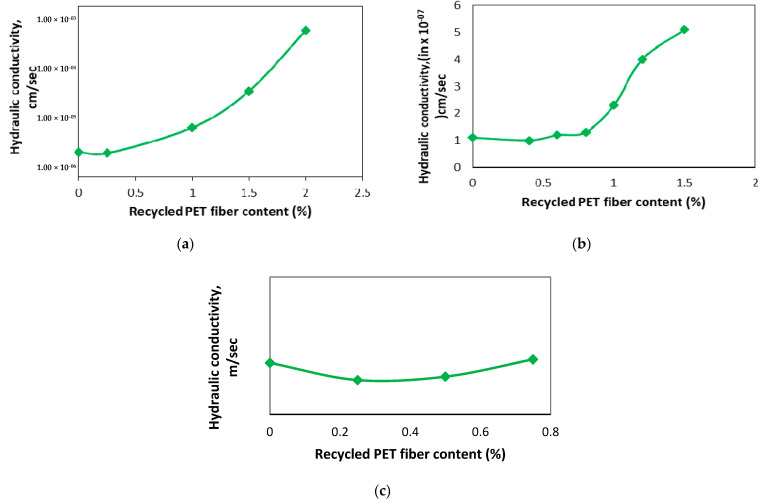
Variation in crack reduction ratio with different recycled PET fiber contents: (**a**) [80], (**b**) [99], and (**c**) [100].

Miller and Rifai [80] examined the relationship between recycled PET fiber percent content and hydraulic conductivity. The results show that the hydraulic conductivity of the soil is influenced by the amount of fibers present, typically increasing with higher fiber content. The most substantial increase in permeability is observed for fiber percentage exceeding 1%, as shown in Figure 13a. The data presented in Figure 13b illustrate the changes in soil hydraulic conductivity as the soil is combined with recycled PET. Initially, there is a decrease in the value of hydraulic conductivity when the additive is introduced, up to a concentration of 0.5%. However, beyond that point, the hydraulic conductivity starts to increase with higher percentages of PET, up to 1.5%, indicating an upward trend in the relationship between the additive and hydraulic conductivity [99]. For silty soil, there was a slight reduction in hydraulic conductivity when the fiber content reached 0.25% and 0.50%, as presented in Figure 13c. However, when the fiber percentage increased to 0.75%, the hydraulic conductivity was nearly indistinguishable from that of the soil without any reinforcement [100]. An increase in the value of the hydraulic conductivity of soil indicates that the soil’s ability to transmit water or other fluids through its pore spaces has improved [101,102].

### 6.9. Influence of Recycled PET Fibers on Soil’s Elastic Modulus

**Figure 14:**
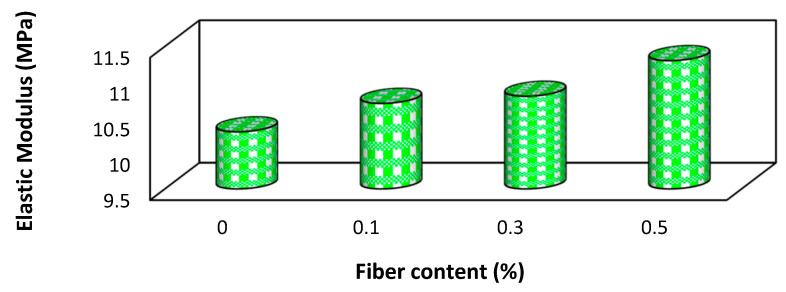
Variation caused by recycled PET fibers in elastic modulus of soil [72].

Figure 14 illustrates that there is an increase in the elastic modulus as the content of recycled PET fiber increases in expansive soil. This indicates that the addition of recycled PET fibers contributes to the stiffness and rigidity of the material [72]. The higher elastic modulus values signify an enhanced ability of the material to resist deformation under applied stress, highlighting the reinforcing effect of the PET fibers on the geotechnical material [103,104].

### 6.10. Influence of Recycled PET Fibers on Soil’s Unconfined Compressive Strength

**Figure 15:**
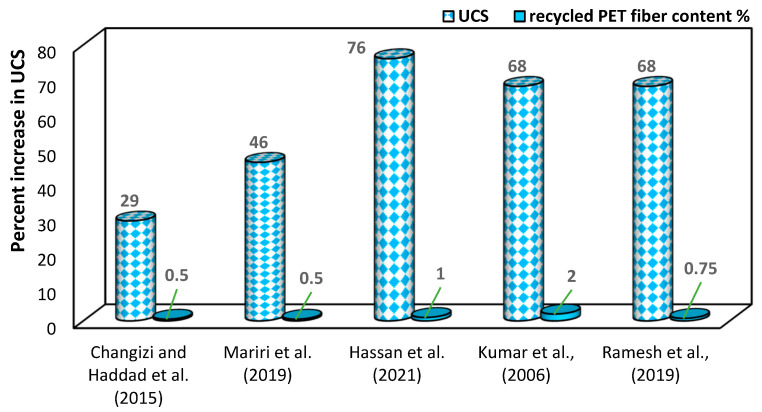
Variation caused by recycled PET fibers in UCS value of soil [71,72,105,107,109].

In soil stabilization, the use of recycled PET fiber contributes to an improvement in the unconfined compressive strength (UCS) value. This enhancement in UCS indicates a strengthening effect on the stabilized soil, making it more resistant to compressive forces. Mariri, Ziaie Moayed and Kordnaeij [71,72] examined a progressive rise in UCS as the PET content increased, reaching a peak at 0.5% in silty soil. According to Hassan, et al. [105] research, with an increase in the amount of recycled PET fiber by up to 1% in the clayey soil, the unconfined compressive strength (UCS) experienced a remarkable enhancement of up to 76% in clayey soil. The strength of clay shows a significant improvement ranging from 50% to 68% with the incorporation of recycled PET fibers of 3 mm in size, comprising 0.5% to 2% of the total mixture [106]. As the percentage of PET fiber reinforcement increased, the UCS value demonstrated a corresponding increase, reaching its optimal point at 10% reinforcement. At this point, the UCS showed the highest improvement of 11% compared to its initial value of 325 kN/m^2^. However, the strength gradually declined once the reinforcement reached 20% [107]. Furthermore, Bhardwaj and Walia [88] observed that for a constant length of PET fibers, the unconfined compressive strength (UCS) value increases by up to 68% as the fiber percentage rises to 0.75%. However, beyond this point, with further increases in fiber content, the UCS value starts to decrease. According to Emmanuel, et al. [108], with the increase in recycled PET fiber content from 0.5% to 1.9%, there is an increase in UCS value. From the above observation, it is seen that clayey soils show maximum improvement in UCS with the incorporation of PET fibers of more than 1%, while silty soils show maximum improvement in UCS with the incorporation of PET fibers of less than 1%. A comparison of the UCS results obtained by previous research is shown in Figure 15.

### 6.11. Influence of Recycled PET Fibers on Soil’s Cohesion and Frictional Angle

The introduction of recycled PET strips into soil has been found to have a significant effect on both cohesion and friction angle, as shown in Table 2 [110,111]. By incorporating these strips into the soil matrix, cohesion is enhanced, leading to increased stability and improved shear strength [112]. Furthermore, the presence of recycled PET strips also influences the soil’s friction angle. The strips create additional contact points and roughness within the soil matrix, increasing the inter-particle resistance to sliding [113]. This enhanced friction angle contributes to the soil’s resistance against shear forces and reduces the likelihood of soil movement or failure. Sarli, Hadadi and Bagheri [81] conducted a study investigating the impact of recycled PET fibers ranging from 0.5 to 1.5% on the cohesion and angle of internal friction of silty clayey sand. The findings revealed that as the percentage of fibers increased, both the cohesion and internal friction angle of the silty clayey soil increased. Similarly, Changizi and Haddad [72,86] conducted research using recycled PET fibers ranging from 0.1 to 0.5% on fat clay soil and clayey sand and observed a similar effect on cohesion and friction angle value. Additionally, Ahmadi, et al. [114] examined the effects of recycled PET fiber content ranging from 0.2 to 1% on clay and found that the presence of fibers led to a gradual increase in both cohesion and internal friction angle values of the soil.

## 7. Applications of Recycled PET Strips in Soil Stabilization

Recycled geotextile PET strips have found valuable applications in soil stabilization, where they contribute to improving the engineering properties of soil [115,116]. One notable use is in the reinforcement of slopes and embankments. With the incorporation of PET strips into the soil, a grid-like structure is created that enhances the soil’s tensile strength and stability [117]. This reinforcement prevents erosion and reduces the risk of landslides, providing long-term stability for slopes and embankments [118]. In road construction, recycled PET strips are used to strengthen subgrade and sub-base layers, enhancing load-bearing capacity and minimizing cracking [115,119]. Besides their functional benefits, the use of recycled PET strips promotes sustainability by repurposing waste materials and reducing the environmental impact associated with virgin materials [120,121]. Overall, incorporating recycled PET strips into soil stabilization techniques offers a range of advantages, including improved strength, erosion control, and environmental sustainability, as discussed below.

### 7.1. Influence of Recycled PET Strips on Soil’s Dry Density

When considering the effect of PET strips on the dry density of soil, studies and research have shown that there is typically no substantial impact [115,122]. The addition of PET strips is primarily aimed at reinforcing the soil and improving its mechanical properties, rather than altering its density characteristics. Dry density is mainly influenced by compaction efforts, water content, and the soil’s particle size distribution [123]. PET strips, being lightweight and of relatively low volume compared to the soil mass, do not significantly contribute to changes in the overall density. Their presence may slightly affect the void ratio and porosity, but the overall impact on dry density is minimal. It is important to note that other factors such as compaction energy and compaction moisture content have a more significant influence on achieving the desired dry density.

### 7.2. Influence of Recycled PET Strips on Soil’s Normal–Shear Stress Characteristics

**Figure 16:**
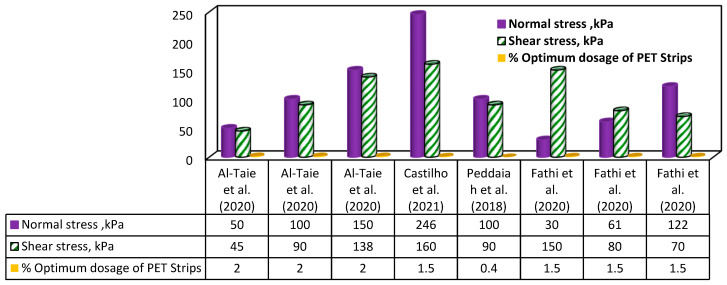
Variation caused by recycled PET strips in normal–shear stress properties of soil [87,115,126,127].

The incorporation of recycled PET strips into soil has been found to affect the normal–shear stress characteristics of the soil, as shown in Figure 16 [124]. The presence of PET strips can enhance the soil’s shear resistance, leading to increased shear strength and improved stability [125]. In the study of Al-Taie, Al-Obaidi and Alzuhairi [87], the influence of incorporating 2% recycled PET fibers on the shear stress of poorly graded soil was investigated. The results showed that as the normal stress (σ_n_) increased at a constant PET fiber content, the shear stress value of the poorly graded soil also increased. Castilho, et al. [126] conducted a similar study on both sandy and clayey soil, using a 1.5% recycled PET fiber content and σn value of 246 kPa. The findings indicated an improvement in the shear stress value of the soil. Peddaiah, Burman and Sreedeep [115] examined the effect of 0.4% PET strip content on sandy soil with a σn value of 100 kPa, while Fathi, et al. [127] studied the impact of a 1.5% PET strip fiber content on sandy soil with varying ranges of σn (30, 61, and 122 kPa) and observed a rise in the shear stress of the soil. This improvement is observed across different soil types and normal stress levels, suggesting that recycled PET strips can be a valuable solution for improving the properties of soils.

### 7.3. Influence of Recycled PET Strips on Soil’s CBR

**Figure 17:**
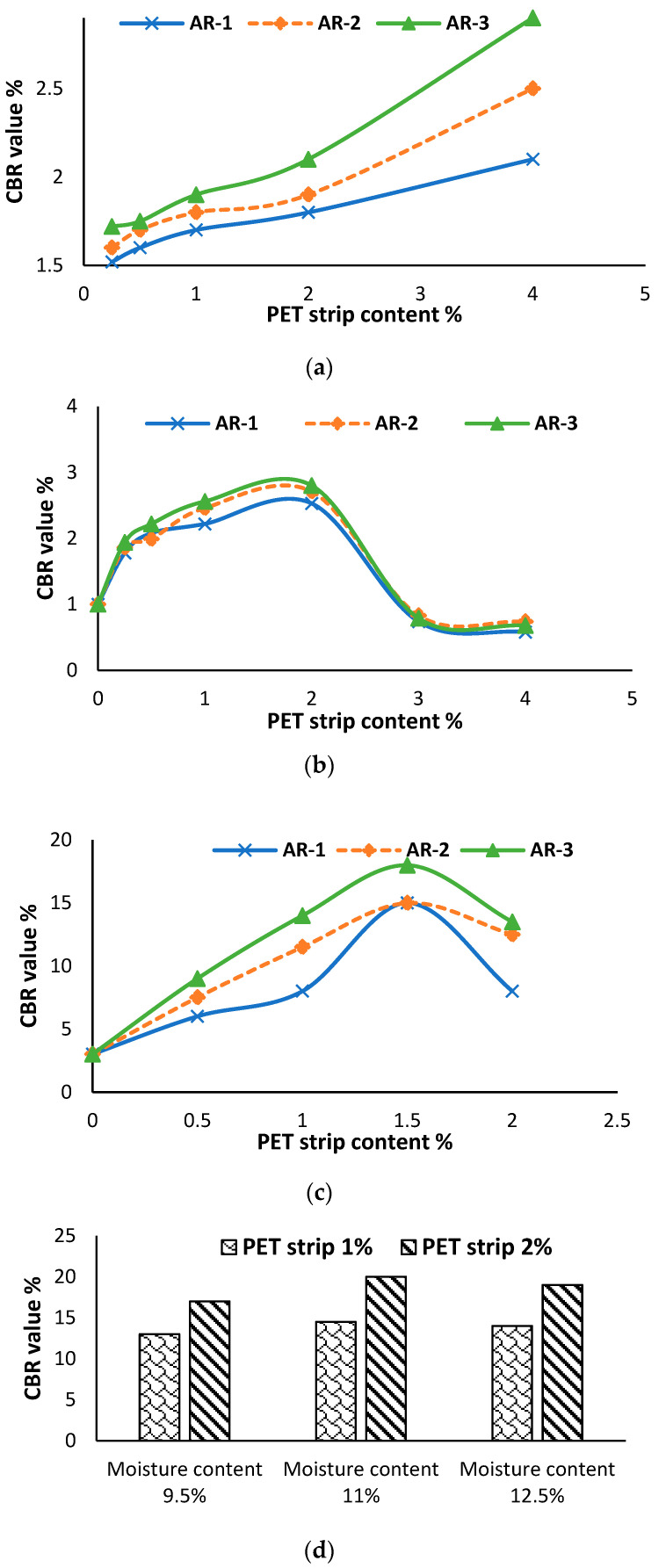
Variation caused by recycled PET strips in CBR value of soil: (**a**) [128], (**b**) [129], (**c**) [130], and (**d**) [83].

Figure 17a–c illustrate the highest enhancement in the CBR resulting from the inclusion of recycled PET strips by using the aspect ratios (ARs) of 1, 2, and 3, while Figure 17d shows the highest improvement in the CBR resulting from the inclusion of recycled PET strips with the moisture contents of 9.5, 11, and 12.5%. The maximum enhancement in the CBR was observed when utilizing 4% PET strip content with the ARs of 1, 2, and 3 [128]. Malicki, Górszczyk and Dimitrovová [83] conducted a study to investigate how moisture content and the addition of recycled PET strips impact the enhancement of the CBR. The highest CBR value was attained with 11% moisture content and 2% PET strip content. Sinha, et al. [129] stated that the highest CBR value can be achieved by incorporating 2% recycled PET strip content. However, the findings of Rawat and Kumar [130] suggest that a maximum CBR value is attained with 1.5% PET strip content at a penetration depth of 5 mm. Amena and Kabeta [119] conducted research to investigate how the inclusion of recycled PET strips and marble dust affects the CBR swell value of expansive soil. Amena and Kabeta [119] found that as the content of marble dust and PET strips increased, there was a noticeable decrease in the CBR swell value. According to the findings of Peddaiah, Burman and Sreedeep [115], an improvement in the value of the CBR was observed when the PET strip percent content was increased up to 0.4%. However, beyond this threshold, a decrease in the CBR value was observed. Niyomukiza, et al. [131] performed the CBR test on soil samples soaked for four days and noted an increase in the CBR value up to a recycled PET content of 0.3%. However, beyond this point, the CBR value started to decrease. Through analysis of sandy and clayey soil, Marçal, et al. [132] investigated the impact of recycled PET strips on the CBR value. Marçal, Lodi, Correia, Giacheti, Rodrigues and McCartney [132] observed a significant improvement in the CBR, from 27% to 47%, when using recycled PET strip content in sandy soil. In contrast, in clayey soil, the value of the CBR decreased from 20% to 18% with the use of recycled PET strips. Therefore, the use of recycled PET strips in soil can potentially affect the CBR value. Incorporating recycled PET strips into the soil can improve its strength and stability, resulting in an increase in the value of the CBR [133,134].

### 7.4. Influence of Recycled PET Strips on Soil’s Indirect Tensile Strength

**Figure 18:**
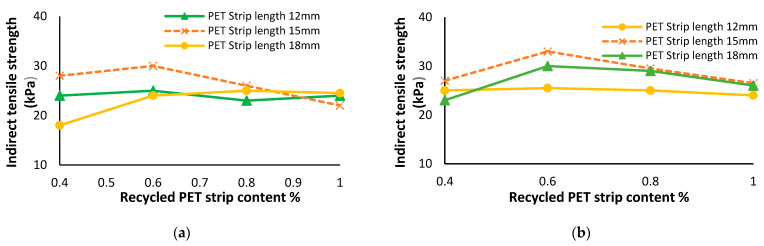
Variation caused by recycled PET strips in indirect tensile strength of soil [137]: (**a**) PET strip length 3 mm and (**b**) PET strip length 6 mm.

The incorporation of recycled PET strips in soil has a beneficial impact on the soil’s indirect tensile strength. This reinforcement leads to increased resistance to cracking and deformation, resulting in improved stability and performance of the soil [135]. Khoury, et al. [136] assessed the effect of recycled PET strips on the indirect tensile strength of silty soil. Their study found that the inclusion of recycled PET strips resulted in a notable enhancement, with the indirect tensile value experiencing an increase of up to 25%. In Figure 18, the results of the tensile strength test indicate that the addition of recycled PET strips to the soil specimens resulted in an improvement in their tensile strength. The specimens reinforced with PET strips of 3 mm and 6 mm in width and of 12 mm, 15 mm, and 18 mm in length were studied, with recycled PET strip content ranging from 0% to 1%. The findings demonstrate that increasing the recycled PET content from 0.4% to 0.6% led to a significant enhancement in the tensile strength of the reinforced soil specimens [137].

### 7.5. Influence of Recycled PET Strips on Soil’s Resilience Modulus

**Figure 19:**
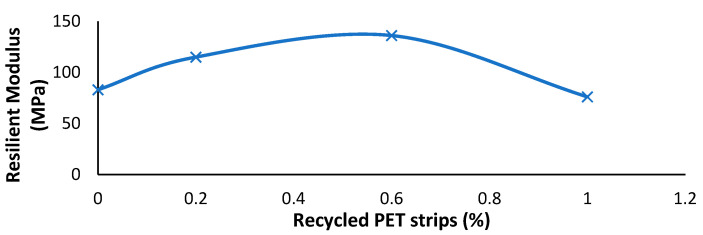
Variation caused by recycled PET strips in resilience modulus of soil [139].

Recycled PET strips are utilized for soil stabilization, enhancing resilience modulus. These strips reinforce the soil, increasing its load-bearing capacity and overall stability [138]. With the incorporation of PET strips, the soil’s resilience modulus is significantly improved, ensuring long-term durability. El-Badawy [139] research demonstrates that the resilience modulus exhibits a consistent increase with increasing content of PET strips, reaching a peak improvement of 56%. However, once the PET strip content surpasses 0.6%, there is a subsequent decrease in the resilience modulus, as depicted in Figure 19.

### 7.6. Influence of Recycled PET Strips on Soil’s UCS Value

**Figure 20:**
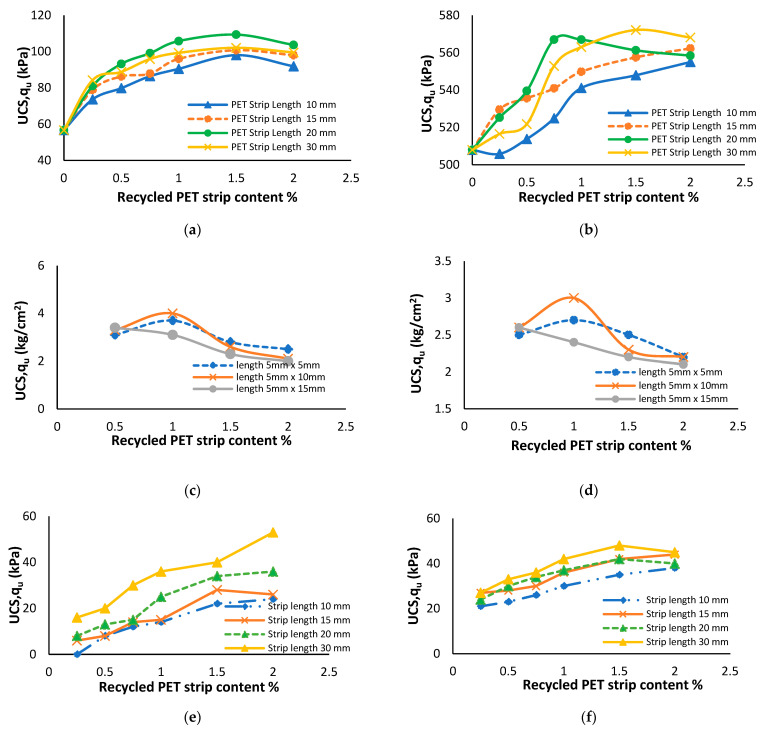
Variation caused by recycled PET strips in UCS value of soil: (**a**) for sandy soil, (**b**) for clayey soil [126], (**c**) for clayey soil with 20% sand, (**d**) for clayey soil [143], (**e**) for sandy soil, and (**f**) for clayey soil [132].

Using PET strips in soil for enhancing UCS offers several advantages. PET acts as a reinforcing material, improving the strength and stability of the soil. The addition of recycled PET strips enhances the UCS value of soil, increasing its load-bearing capacity and performance [140,141]. Figure 20a,b illustrate the enhancement in UCS value resulting from the incorporation of recycled PET strips in both clayey and sandy soil. The most significant improvement in UCS value for both sand and clayey soil was observed when 1.5% of recycled PET strip content was added [126,142]. The results depicted in Figure 20c,d demonstrate the increase in UCS value caused by the addition of 1% PET strips to clayey soil and clayey soil with 20% sand [143]. According to Roustaei, Tavana and Bayat [137], the specimens exhibited an increasing trend in UCS as the content of PET strips increased until reaching 0.8%, after which the UCS began to decline. Among all the PET strip lengths tested, the specimens with 0.8% PET strip content achieved the highest UCS value. Kabeta [140] conducted a study on the influence of recycled PET strips (ranging from 0% to 0.4%) on soft clay soil. The research revealed a significant increase in the UCS value of soil, reaching up to 138% improvement with the use of 2 mm long PET strips. A gradual increase in the UCS value was observed when utilizing PET strip lengths of 10 mm, 15 mm, 20 mm, and 30 mm and varying the recycled PET strip content from 0.25% to 2% in sandy and clayey soils, as shown in Figure 20e,f [132].

### 7.7. Influence of Recycled PET Strips on Soil’s Cohesion and Internal Frictional Angle

**Figure 21:**
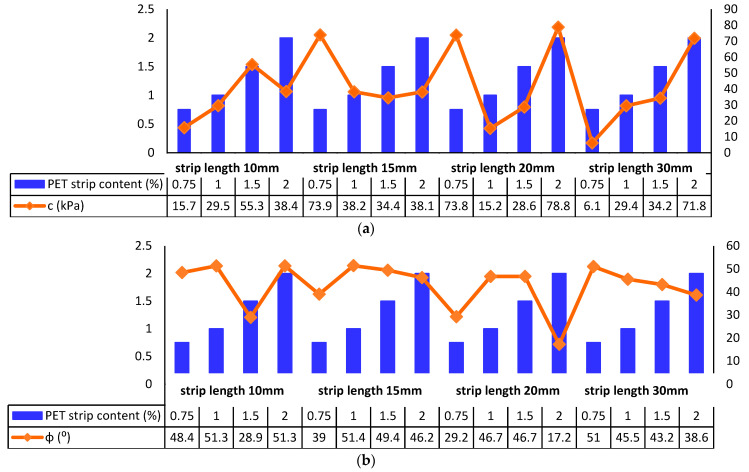
Variation caused by recycled PET strips in: (**a**) cohesion and (**b**) frictional angle value of soil [142].

The inclusion of recycled PET strips has been found to have a notable impact on both cohesion and the frictional angle of soil. Cohesion, which represents the shear strength of soil particles, tends to increase with the addition of recycled PET strips. On the other hand, the frictional angle, which signifies the resistance to sliding between soil particles, is observed to decrease when recycled PET strips are incorporated. These effects suggest that the presence of recycled PET strips alters the mechanical properties of soil, enhancing its cohesive strength while reducing its resistance to sliding. The findings of Marçal, Lodi, Correia, Giacheti, Rodrigues and McCartney [132] indicate that the utilization of recycled PET content in sandy soil results in an increase in the frictional angle by approximately 1.18%. Similarly, for clayey soil, the frictional angle increases by approximately 1.47% with the addition of recycled PET content. Additionally, there is an increase in the cohesion value of approximately 2.26% for sandy soil and 0.86% for clayey soil when incorporating recycled PET strip content. Silveira, Lodi, Correia, Rodrigues and Giacheti [142] investigated the influence of incorporating recycled PET strips, ranging from 0.75% to 2% by weight, into cement-treated lateritic soil. The experiment included different lengths of PET strips (10, 15, 20, and 30 mm). However, the results, depicted in Figure 21, demonstrated no consistent changes in the values of cohesion and frictional angle.

## 8. Environmental Implication of PET Addition to Soil

The major environmental improvement concerns when adding PET to soil stabilization include several key factors. Potential microplastic pollution arises from the degradation of PET fibers, which can harm wildlife and enter the food chain. Chemical leaching of toxic additives and byproducts into the soil and groundwater poses risks to soil health, biodiversity, and human health [144]. The long-term persistence of non-biodegradable PET in the environment complicates remediation efforts and raises sustainability issues [145]. However, the use of recycled PET in soil stabilization also offers environmental benefits. It aids in enhanced waste management by repurposing plastic waste, thereby reducing the volume of plastic in landfills and oceans. This practice supports the circular economy by conserving natural resources and energy compared to producing new materials [146]. Additionally, PET reinforcement can improve soil stability, bearing capacity, and infrastructure durability, leading to more sustainable construction practices. Balancing these concerns requires effective monitoring and regulation to minimize negative impacts. Ongoing research into biodegradable alternatives and sustainable practices is essential. Implementing best practices for PET use in geotechnical applications, including proper installation and limiting the amount used, can help maximize environmental benefits while mitigating potential harms [147].

## 9. Economic Implication of PET Addition to Soil

The addition of PET to soil stabilization offers various economic implications that span across cost savings, waste management efficiency, and market opportunities. Firstly, by utilizing recycled PET fibers and strips, construction projects can achieve cost savings on raw materials. The reduction in the need for new materials translates to lower procurement costs, contributing to overall project affordability [148]. Moreover, the efficient management of plastic waste through PET incorporation reduces municipal waste management expenses, as less material is destined for landfills or costly recycling processes. This efficiency not only benefits construction projects directly but also alleviates the financial strain on waste management systems, potentially leading to broader economic savings [149]. Secondly, the market demand for recycled materials is bolstered by the adoption of PET in soil stabilization. As the construction industry increasingly values sustainable practices, the demand for recycled PET grows, stimulating growth within the recycling sector. This, in turn, creates economic opportunities for recycling industries and encourages innovation in recycling technologies [150]. Additionally, the extended lifespan of infrastructure resulting from PET reinforcement reduces maintenance costs over time, contributing to long-term economic benefits [151]. While there may be initial investments in compliance and research, the overall economic outlook for PET addition to soil stabilization is promising, with the potential for significant cost savings and market growth.

**Figure 22:**
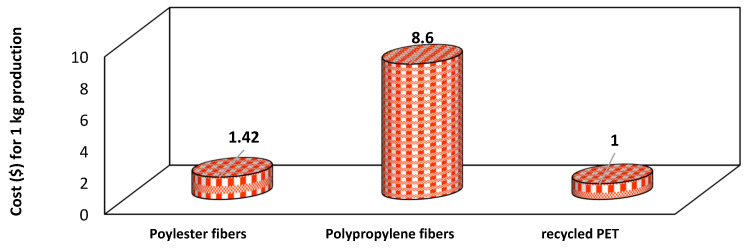
Comparative cost analysis of different fibers [87].

As shown in Figure 22, Al-Taie, Al-Obaidi and Alzuhairi [87] conducted a comparative cost analysis for the production of 1 kg of polypropylene fiber, polyester fibers, and recycled PET. The findings indicated that the production cost for recycled PET was the lowest, approximately USD 1, in comparison to the other materials. This demonstrates that utilizing recycled PET is a viable and favorable option for the construction industry. The availability and cost-effectiveness of recycled PET make it an attractive option for engineers and project developers. By incorporating recycled materials, construction projects can lower expenses, enhance sustainability ratings, and potentially gain recognition for environmentally responsible practices.

## 10. Conclusions

Considering the various studies conducted by researchers on the usage of recycled PET fibers and strips for soil stabilization and analysis of different soil properties, the main outcomes of the review are as follows:

According to the majority of researchers, there exists an optimal content of recycled PET fibers and strips that leads to the maximum improvement in soil properties. However, this optimal percentage varies depending on factors such as soil classification and specific soil types. Through the examination of the impact of recycled PET fibers on soil stabilization, several studies have demonstrated that the enhancement in stress–strain response is primarily recognized as the reinforcing effect of the PET fibers. These fibers assist in the uniform distribution of stresses, resulting in a more desirable and controlled deformation response.

Numerous studies have consistently demonstrated that the addition of recycled PET fibers and strips does not considerably impact the dry density of soil. Instead, the primary objective of incorporating PET fibers and strips is to strengthen the soil and enhance its mechanical properties, rather than altering its density characteristics. Dry density is primarily influenced by the compaction efforts employed during construction.

However, the addition of recycled PET fibers up to 1% and strips up to 2% has been shown to have a positive and significant impact on the normal and shear stress values of the soil, indicating mechanical improvements in soil stabilization. This reinforcement effect contributes to the enhanced performance of the soil under loading conditions.

Furthermore, many researchers have examined the influence of increasing recycled PET content on the CBR value. In general, the value of the CBR gradually increases with the addition of PET fibers up to 1.2% and strips up to 4% with an aspect ratio of 3. Beyond this optimum, the CBR value starts to decrease. It is worth noting that a study performed by Kumar, et al. [109] on black cotton soil observed an initial decrease in the CBR value, followed by a gradual increase as the content of recycled PET fibers increased up to 3%.

Various research studies have shown that the addition of recycled PET fibers up to 2% and 0.5% has a positive effect on the hydraulic conductivity and resilience modulus of clayey soil, leading to their increased values. Additionally, many researchers have reported an improvement in the UCS of soil by incorporating recycled PET fibers and strips. The majority of studies have found that the maximum UCS value is achieved with a 1% content of recycled PET fibers and 1.5% of PET strips in clayey soil, although there may be variations depending on the soil type. When it comes to the cohesion and internal friction angle of soil, the addition of recycled PET fibers has been found to gradually increase both parameters. The extent of this increase depends on the specific soil type. The maximum improvement is seen in clayey soil when using PET fibers up to 1% and PET strips up to 2% with a length of 20 mm. In the case of recycled PET strips, the values of cohesion and friction angle of the soil gradually increase, considering both the soil type and the length of the strips.

## Figures and Tables

**Table 1:** Thermal, mechanical, and physiochemical properties of PET [42].

		PET	
		Semi-Crystalline	Amorphous
Thermal properties			
T_g_ (°C)		68–80	60–84
T_max_ (°C)		115–120	55–65
T_mould_ (°C)		125–145	20–30
T_m_ (°C)		255–265	-
Mechanical properties			
E (GPa)		2.8–3.1	2.8–3.0
ε_b_ (%)		65–75	280–320
σ_max_ (MPa)		70–75	55–60
Physiochemical properties			
Density (g/cm^3^)		1.37–1.40	1.29–1.39
Permeability, 25 °C (cm^3^ mm)	CO_2_	14.0	15.7
	O_2_	1.2–2.8	1.2–2.8
Transparency		Opaque	Transparent

Abbreviations: Transition temperature (T_g_), maximum service temperature (T_max_), mold temperature (T_mould_), melting temperature (Tm), Young’s modulus (E), elongation at break (ε_b_), maximum stress (σ_max_).

**Table 2:** Variation caused by recycled PET fibers in cohesion and frictional angle of soil.

References	Specimen	1	2	3	4
Silty clayey [81]	PET fiber content (%)	0	0.5	1	1.5
	Cohesion, c, (kPa)	11	18	24	32
	Angle of internal friction, ϕ, (°)	24	26	28	31
Fat clay [72]	PET fiber content (%)	0	0.1	0.3	0.5
	Cohesion, c, (kPa)	38	56	59	64
	Angle of internal friction, ϕ, (°)	13.5	14.6	19.3	23.3
Clayey sand [86]	PET fiber content (%)	0	0.1	0.3	0.5
	Cohesion, c, (kPa)	30.92	39.93	43.05	46.88
	Angle of internal friction, ϕ, (°)	27.04	32.85	34.51	35.9
Clay [114]	PET fiber content (%)	0	0.2	0.5	1
	Cohesion, c, (kPa)	62	72.4	95.2	97.4
	Angle of internal friction, ϕ, (°)	17.2	21.8	25.2	27

## Data Availability

The data presented in this study are available upon request due to privacy.
